# Suppression of human T cell proliferation by the caspase inhibitors, z-VAD-FMK and z-IETD-FMK is independent of their caspase inhibition properties

**DOI:** 10.1016/j.taap.2012.09.002

**Published:** 2012-11-15

**Authors:** C.P. Lawrence, S.C. Chow

**Affiliations:** a Medical Research Council Toxicology Unit, Hodgkin Building, Lancaster Road, University of Leicester, Leicester LE1 9HN, UK; b School of Science, Monash University Sunway Campus, Jalan Lagoon Selatan, Bandar Sunway, 46150 Selangor Darul Ehsan, Malaysia

**Keywords:** T lymphocytes, z-VAD-FMK, z-IETD-FMK, caspases, T cell activation

## Abstract

The caspase inhibitors, benzyloxycarbony (Cbz)-l-Val-Ala-Asp (OMe)-fluoromethylketone (z-VAD-FMK) and benzyloxycarbonyl (Cbz)-Ile-Glu (OMe)-Thr-Asp (OMe)-FMK (z-IETD-FMK) at non-toxic doses were found to be immunosuppressive and inhibit human T cell proliferation induced by mitogens and IL-2 in vitro. Both caspase inhibitors were shown to block NF-κB in activated primary T cells, but have little inhibitory effect on the secretion of IL-2 and IFN-γ during T cell activation. However, the expression of IL-2 receptor α-chain (CD25) in activated T cells was inhibited by both z-VAD-FMK and z-IETD-FMK, whereas the expression of the early activated T cell marker, CD69 was unaffected. During primary T cell activation via the antigen receptor, both caspase-8 and caspase-3 were activated and processed to their respective subunits, but neither caspase inhibitors had any effect on the processing of these two caspases. In sharp contrast both caspase inhibitors readily blocked apoptosis and the activation of caspases during FasL-induced apoptosis in activated primary T cells and Jurkat T cells. Collectively, the results demonstrate that both z-VAD-FMK and z-IETD-FMK are immunosuppressive in vitro and inhibit T cell proliferation without blocking the processing of caspase-8 and caspase-3.

## Highlights

► Caspase-8 and caspase-3 were activated during T cell activation and proliferation. ► T cell proliferation was blocked by caspase inhibitors. ► Caspase activation during T cell proliferation was not block by caspase inhibitors.

## Introduction

The important role of caspases, particularly caspase-8 in T cell activation and proliferation is now firmly established (Chun et al., 2002). However, much of the early evidence for the role of caspase involvement in mitogen-induced T cell proliferation came largely from studies using peptidyl-FMK caspase inhibitors, which were shown to markedly decrease mitogen-induced T cell proliferation (Alam et al., 1999; Boissonnas et al., 2002; Falk et al., 2004; Kennedy et al., 1999; Mack and Hacker, 2002). Besides blocking mitogen-induced T cell proliferation (Chun et al., 2002; Falk et al., 2004) these caspase inhibitors were also shown to reduce the expression of the α-subunit of the IL-2 receptor, CD25 and inhibit IL-2 secretion in activated T cells (Falk et al., 2004; Kennedy et al., 1999).

All peptidyl-FMK caspase inhibitors contain a peptide sequence based on the target cleavage sequence of the substrate and act as competitive inhibitors by mimicking the substrate. These enzymes recognise a sequence of four amino acids in the substrates, designated P4-P3-P2-P1 and cleave substrates after an Asp residue at P1 (Yuan et al., 1993). All peptide-based caspase inhibitors used to date consist of a peptide sequence culminating in an Asp residue (Garcia-Calvo et al., 1998). The requirement for specific amino acid residues at the other positions varies with members of the caspase family. This enables more specific caspase inhibitors to be developed by exploiting the different substrate specificities (Garcia-Calvo et al., 1998; Thornberry et al., 1997). Conjugated to the peptide sequence of the caspase inhibitor is a halomethylketone, such as fluoromethylketone (FMK), which form irreversible covalent bonds with the S-H group of the cysteine residue in the caspase active site (Caserta et al., 2003; Garcia-Calvo et al., 1998). Finally, the amino-terminal group, usually a benzyloxycarbonyl (z) or acetyl (Ac) group, enhances the cell permeability of the inhibitor by increasing the hydrophobicity of the compound (Van Noorden, 2001).

These peptidyl-FMK caspase inhibitors are extremely useful tools and were used extensively in apoptosis research to elucidate the role of caspases during apoptotic cell death. However, accumulating evidence also suggests that these inhibitors may not be as specific as originally anticipated. For instance, the widely-used broad-spectrum caspase inhibitor, z-VAD-FMK, has also been shown to inhibit other enzymes, such as the lysosomal cysteine protease, cathepsin B (CatB) (Schotte et al., 1999), peptide:*N*-glycanase (PNGase) (Misaghi et al., 2006) and picornaviral 2A proteinases (Deszcz et al., 2004). In addition, the caspase-8 inhibitor, z-IETD-FMK also inhibited picornaviral 2A proteinases (Deszcz et al., 2004). Some of the non-specific effects of these caspase inhibitors (z-VAD-FMK and z-IETD-FMK) may account for their inconsistencies in blocking T cell activation and proliferation as reported in a number of early studies (Mack and Hacker, 2002; Zapata et al., 1998). In addition, the caspase-3-selective inhibitor, z-DEVD-FMK, which blocked T cell proliferation (Alam et al., 1999), was subsequently shown to have little effect in other studies (Boissonnas et al., 2002; Kennedy et al., 1999; Mack and Hacker, 2002).

In the present study we examined the immunosuppressive properties of the peptidyl-FMK caspase inhibitors, z-VAD-FMK and z-IETD-FMK, and determined whether their inhibition of mitogen-induced T cell proliferation is due to the blocking of caspase processing during T cell activation. Our results showed that both caspase inhibitors readily block T cell proliferation induced by mitogens as well as IL-2. However, these peptidyl-FMK caspase inhibitors had little effect on the processing of caspase-8 and caspase-3 to their respective subunits during T cell activation although they efficiently blocked caspase activation during apoptosis. Taken together, these results suggest that the inhibition of T cell proliferation mediated by these caspase inhibitors is independent of their caspase inhibition properties.

## Materials and Methods

#### Reagents

Benzyloxycarbonyl-Val-Ala-Asp-(O-methyl)-fluoromehylketone (z-VAD-FMK), benzyloxycarbonyl-Ile-Glu-Thr-Asp-fluoromethylketone (IETD-FMK) and benzyloxycarbonyl-Phenyl-Alanyl-acid-fluoromethylketone (z-FA-FMK) were purchased from ICN (USA). Monoclonal antibody (mAb) against CD3 (clone OKT3) was purified from hybridoma (ATCC) culture supernatants and anti-CD28 mAb was purchased from R & D (UK). Goat-anti caspase-8 was from Santa Cruz Biotechnology (USA) and rabbit anti-caspase-3 was generous gift from Xiao-Ming Sun, MRC Toxicology Unit (UK). FITC-conjugated anti-CD25 and RPE-conjugated anti-CD69 were acquired from Transduction Laboratories (UK) and Dako (UK), respectively. Recombinant Fas ligand (FasL), anti-Flag and anti-PARP were obtained from Alexis Biochemicals (UK). [^3^H]-thymidine was obtained from Amersham (UK) and phytohaemaglutinin (PHA) was purchased from Sigma (UK). MACS columns and MACS beads conjugated with anti-CD4 and anti-CD8 were obtained from Miltenyi Biotec (Germany). Lymphoprep was from Axis-Shield PoCAS (Norway) and RPMI 1640 and FCS were from Gibco (UK). Hoechst 33358 and carboxyfluorescein diacetate succinimidyl ester (CFSE) were from Molecular Probes (USA).

#### Cell isolation

Peripheral venous blood was obtained from normal healthy volunteers and collected into heparinized Vacutainers (Becton Dickinson). Peripheral blood mononuclear cells (PBMCs) were isolated using density gradient centrifugation with lymphoprep. The cells at the interface between the plasma and lymphoprep were collected, washed and re-suspended in RPMI containing 10% (v/v) foetal calf serum (FCS), 10 mM L-glutamine (Invitrogen, UK), penicillin (100 U/ml) and streptomycin (100 μg/ml). The viability of the lymphocyte population obtained via this procedure was routinely > 95% as assessed by trypan blue exclusion assay. Purified CD4^+^ (~ 97%) and CD8^+^ T (~ 98%) cells were isolated from the PBMCs using anti-CD4 and anti-CD8 mAbs conjugated MACS beads.

#### Cell cultures and treatments

PBMCs (5 × 10^6^ cells/ml) or purified CD4^+^ and CD8^+^ T cells (1 × 10^6^ cells/ml) in RPMI 1640 supplemented with 10% FCS were stimulated with either 5 μg/ml PHA, co-stimulated with plate bound 5 μg/ml anti-CD3 (OKT3 mAb) and 2.5 μg/ml anti-CD28 in the absence or presence of caspase inhibitors for various time periods in an atmosphere of 5% CO_2_ in air at 37 °C. Proliferating T cells were derived by activating purified CD4^+^ and CD8^+^ T cells with PHA for 24 h and then reseeded in media supplemented with rIL-2 (25 Units/ml). The activated T cells were cultured for 7 days prior to use. The human leukemic T cell line, Jurkat, clone E6-1 (ATCC) were maintained in logarithmic phase of growth in RPMI 1640 supplemented with 10% FCS and 2 mM L-Glutamine in an atmosphere of 5% CO_2_ in air at 37 °C. To induce apoptosis, Jurkat T cells (1 × 10^6^ cells/ml) or activated T cells (1 × 10^6^ cells/ml) in complete medium were stimulated with recombinant Flag-tagged FasL (100 ng/ml) followed by cross-linking with anti-Flag (1 μg/ml) for 16 h. Apoptotic cells were determined using UV microscopy, annexin V staining and TMRE labelling of mitochondria as previously described (Jayaraman, 2005; Johnson et al., 2000). Cell viability was determined by suspending treated cells in 500 μl ice-cold PBS with 10 μl of 20 μg/ml propidium iodide (PI) and the uptake of PI was analysis using flow cytometry.

#### Cell proliferation and division assays

T cell proliferation following mitogen stimulation was determined using [^3^H]-thymidine incorporation. In brief, PBMCs or purified T cells were seeded at 1 × 10^6^ cells/ml in 96 well plates and stimulated with either PHA (5 μg/ml) or co-stimulated with anti-CD3 mAb (5 μg/ml) and anti-CD28 mAb (2.5 μg/ml) in the presence or absence of caspase inhibitors. The cells were cultured for 72 h with the last 16 h pulsed with [^3^H]-labelled methyl-thymidine (0.037 MBq) prior to harvest onto glass fibre filter mats using a Tomtec automated multi-well harvester (Perkin Elmer Life Sciences, Boston USA). Wallac Betaplate scintillation reagent (Perkin Elmer Life Sciences) was added to the glass fibre filter mats and the radioactivity was determined on a 1450 Microbeta liquid scintillation counter (Perkin Elmer Life Sciences, Boston USA). T lymphocyte division following mitogen stimulation was determined using CFSE labelling of the cells (Lyons and Parish, 1994). In brief, PBMCs were suspended in PBS at a density of 5 × 10^7^/ml and incubated with 5 μM CFSE at 37 °C for 10 minutes. Following incubation with CFSE the labelled PBMCs were washed twice in RPMI to remove excess CFSE. The CFSE labelled cells were treated with mitogens as previously described in the presence or absence of caspase inhibitors. As the T lymphocytes divide, CFSE is sequentially diluted, resulting in a decreased in fluorescence intensity in the cells, which can be followed by flow cytometry.

#### Measurement of secreted IL-2 and IFN-γ in culture supernatants

Following treatments for 24 h, the T cells were removed by centrifugation and the supernatants collected and kept frozen until used. The secreted IL-2 and IFN-γ in the supernatants were detected using the DuoSet ELISA kits from R & D System (UK) according to the manufacturer's instruction.

#### Determination of cell surface CD25 and CD69 expression

Following treatments, PBMCs (5 × 10^5^ cells) were centrifuged down and the supernatants discarded. The cell pellets were re-suspended in 50 μl staining buffer (2% BSA in PBS). FITC-conjugated anti-CD25 (10 μl), RPE-conjugated anti-CD69 (10 μl) or the appropriate fluorochrome-conjugated mouse IgG (isotype control) were added to the cells and incubated on ice for 30 min in the dark. The cells were then washed twice in staining buffer before analyzed immediately by flow cytometry.

#### Nuclear translocation of NF-κB RelA, p65

This is essentially as described previously (Su et al., 2005). Purified T cells (3 × 10^6^ cells) were co-stimulated with anti-CD3 and anti-CD28 for 2 h, washed with cold PBS and fixed with 1 ml paraformaldehyde (4%) for 20 min at room temperature. The cells were permeabilised with PBS containing 3% BSA and 0.2% triton X-100 for 2 min in room temperature. The permeabilised cells were washed twice and resuspended in 100 μl of PBS with 3% BSA and rabbit anti-p65 antibody (1:50 dilution) for 45 min at room temperature. The cells were then washed and incubated with anti-rabbit antibody conjugated with alexa fluor (1:2000 dilutions) and Hoechst 33348 in a final volume of 200 μl for 30 min in the dark. Following this the cells were washed twice and resuspended in 10 μl PBS: glycerol (50/50, vol/vol). The cells were mounted onto slides and viewed using confocal microscopy. Images were randomly acquired from each sample and cells with NF-κB p65 nuclear localization were counted. A minimum of 500 cells was analyzed for each sample.

#### Western Blotting.

Following treatments, 2 × 10^6^ cells were washed in PBS and resuspended in 30 μl lysis buffer (0.1 M NaCl, 1 mM Tris HCl at pH7.6, 1 mM EDTA, 1% Triton-X, 1 mM PMSF). The cells in lysis buffer were taken through 3x freeze/thaw cycles on dry ice. Protein concentration was measured using the Bradford assay (Biorad, Germany). Protein (30 μg) from whole-cell lysates was diluted in loading buffer (2% SDS, 10% Glycerol, 50 mM Tris–HCl pH 6.8, 0.2% Bromophenol Blue and 100 mM DTT) and resolved using SDS-polyacrylamide gel electrophoresis. The polyacrylamide gels used were 7% for PARP and 13% for caspases. The separated proteins were transfer onto Hybond C membrane (Amersham, UK) and probed with antibodies to caspase-8, caspase-3 and PARP. Detection was carried out using chemiluminescence (Amersham).

#### Statistical analysis of the data

The experimental data were analysed using Student's *t* test or One-way analysis of variance followed by Dunnet's test.

## Results

### Effect of z-VAD-FMK and z-IETD-FMK on primary T cell proliferation

**Fig. 1:**
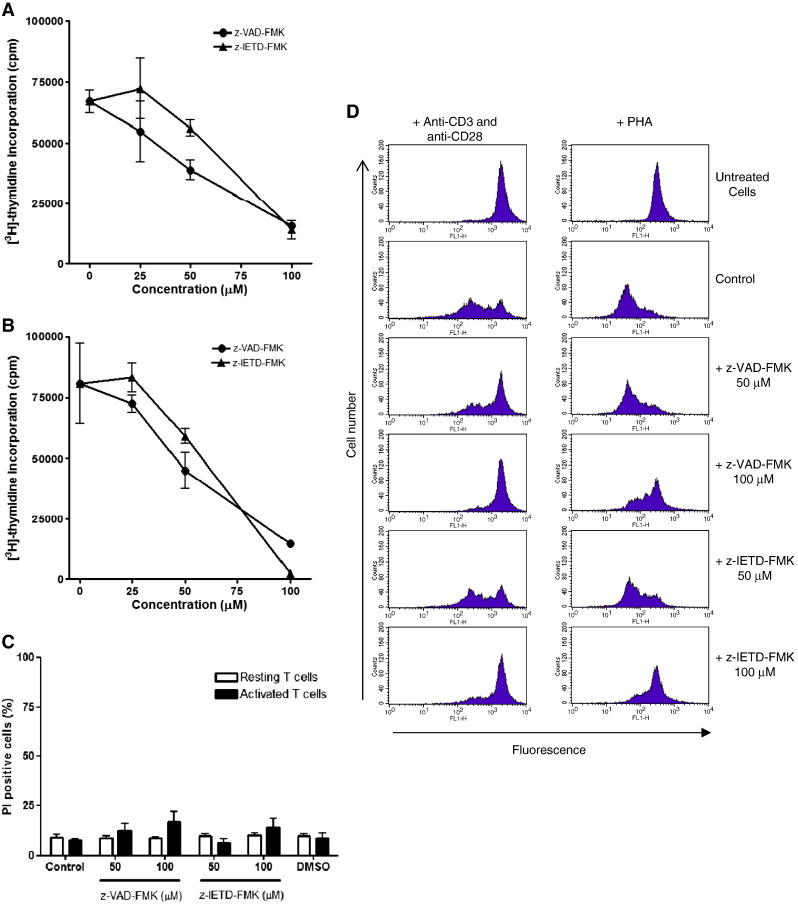
Dose-dependent inhibition of T cell proliferation by peptidyl FMK caspase inhibitors. T cell proliferation in PBMCs co-stimulation with anti-CD3 and anti-CD28 (A) or stimulated with PHA (B) alone in the presence or absence of various concentrations of z-VAD-FMK and z-IETD-FMK. T cell proliferation was assayed using [^3^H]-thymidine incorporation. The toxicity of both caspase inhibitors was assessed after 24 h in resting T cells and after 72 h in activated T cells using PI uptake (C). Activated T cells undergoing cell division (72 h) are measured using CFSE labelling (D). The results are the means ± SEM from three separate experiments (A, B and C), and one representative from three independent separate experiments (D). DMSO was used as the carrier solvent.

In order to determine the immunosuppressive effects of peptidyl-FMK caspase inhibitors on T cell activation, the effects of z-VAD-FMK and z-IETD-FMK on mitogen-induced T cell proliferation were examined. These two inhibitors were chosen because the former is a pan-caspase inhibitor and the latter a preferred caspase-8 inhibitor. As illustrated in Fig. 1A, z-VAD-FMK dose-dependently inhibited T cell proliferation mediated through the co-stimulation with anti-CD3 and anti-CD28. The caspase-8 inhibitor, z-IETD-FMK was less effective at 25 and 50 μM, but inhibited T cell proliferation to a similar extent as z-VAD-FMK at the higher concentration (100 μM). A similar dose-dependent inhibition was seen with these two peptidyl-FMK caspase inhibitors on PHA-induced T cell proliferation (Fig. 1B). Taken together, these data confirmed previous published findings that both z-VAD-FMK and z-IETD-FMK inhibit mitogen-induced T cell proliferation (Alam et al., 1999; Boissonnas et al., 2002). We next examined whether the decreased in [^3^H]-thymidine incorporation in the presence of these caspase inhibitors was due to direct toxicity of these inhibitors. To this end, the cell viability of primary T cells following treatment with the peptidyl-FMK caspase inhibitors was determined. As shown in Fig. 1C, there was no increased in PI uptake in resting T cells after 24 h treatment with z-VAD-FMK or z-IETD-FMK compared to control untreated cells. This suggests that the caspase inhibitors are not toxic to resting T cells. To further rule out toxicity following T cell activation, PI uptake was also examined in activated T cells in the presence of caspase inhibitors. About 9% of control activated T cells took up PI after activation, whereas in the presence of 100 μM of z-VAD-FMK and z-IETD-FMK cell death increased to 18% and 23%, respectively (Fig. 1C). The increase in PI uptake was not significant (*p* > 0.05) suggesting that the marked inhibition of T cell proliferation is unlikely to be due to the toxicity of these inhibitors. To further corroborate the [^3^H]-thymidine incorporation results (Figs. 1A & B) we examined the effect of the caspase inhibitors on T cell division using CFSE labelling (Lyons and Parish, 1994). The sequential dilution of the CFSE dye following cell division can be followed using flowcytometry. As illustrated in Fig. 1D, the cellular fluorescence intensity remained high in resting T cells over 72 h, confirming that the cells were quiescent. In contrast, T cells co-activated with anti-CD3 plus anti-CD28 were dividing as indicated by the sequential decrease in cellular fluorescence intensity. In the presence of z-VAD-FMK, the decrease in cellular fluorescence intensity was markedly inhibited compared with control activated cells, suggesting that cell division was blocked. This effect was more apparent at 100 μM, where nearly all the cells retained a high cellular fluorescence. In contrast, little effect on cell division was seen with 50 μM z-IETD-FMK, but again at 100 μM, cell division was markedly inhibited to similar extent as z-VAD-FMK. Compared with co-stimulation with anti-CD3 plus anti-CD28, more resting T cells undergo cell division following exposure to PHA (Fig. 1D) as illustrated by the marked decrease in fluorescence after 72 h of activation. We observed that z-VAD-FMK at 50 μM had little effect on PHA-induced T cell proliferation and inhibition was only seen at 100 μM. A similar inhibition pattern was seen with z-IETD-FMK, although this inhibitor appeared to be slightly less potent compared with z-VAD-FMK. These data are very much in line with the [^3^H]-thymidine incorporation data indicating that both caspase inhibitiors are capable of inhibiting T cell proliferation induced by anti-CD3 plus anti-CD28 or PHA. DMSO (> 0.1%), which is the carrier solvent for the caspase inhibitors was included in all the studies and was found to have no effect on T cell proliferation (results not shown).

### Effects of peptidyl-FMK caspase inhibitors on interleukin 2 (IL-2) secretions and signalling

**Fig. 2:**
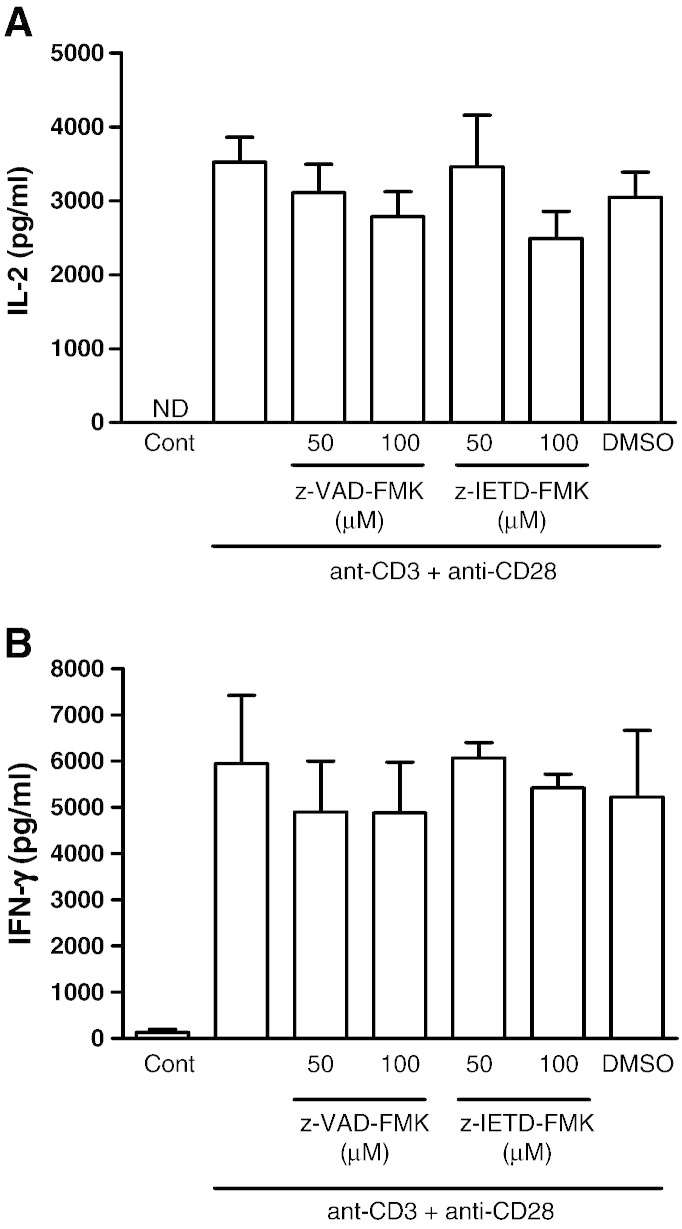
The effect of z-VAD-FMK and z-IETD-FMK on cytokine secretion in activated T cells. Purified CD4^+^ and CD8^+^ T cells were co-stimulated with anti-CD3 and anti-CD28 for 24 h in the presence and absence of z-VAD-FMK or z-IETD-FMK. The amount of IL-2 (A) and IFN-γ (B) secreted into the culture supernatants were determined using ELISA as described in Materials and Methods. Results are the means ± SEM from three independent experiments. ND, not detected. DMSO was used as the carrier solvent.

**Fig. 3:**
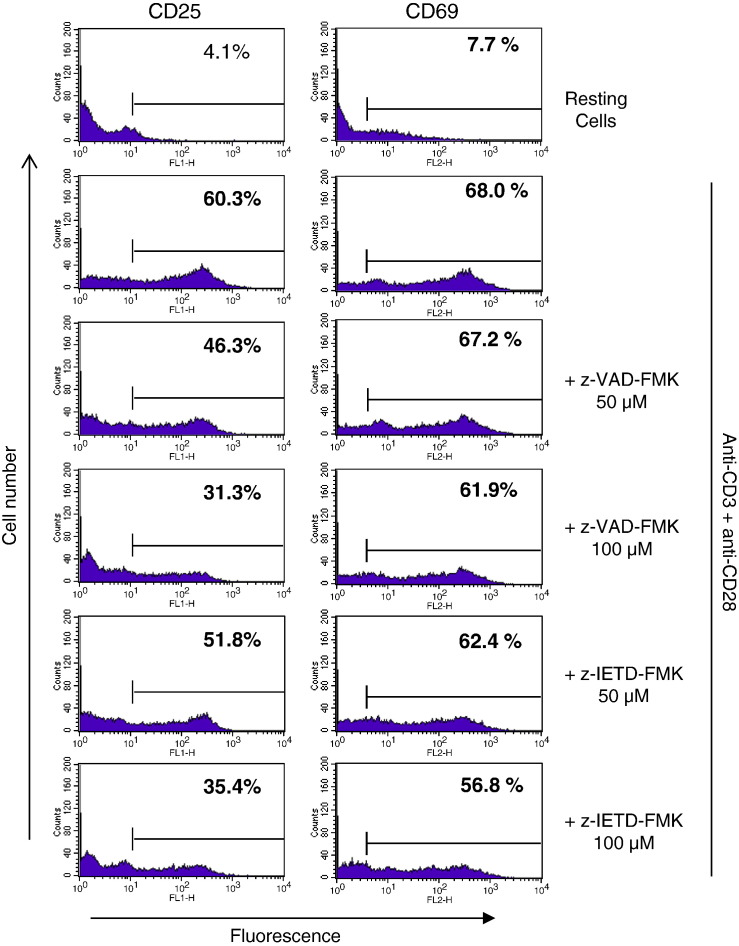
The effect of z-VAD-FMK and z-IETD-FMK on CD25 and CD69 expression in T cells following activation. The T cells in PBMCs were co-stimulated with anti-CD3 and anti-CD28 in the presence and absence of z-VAD-FMK and z-IETD-FMK for 48 h. The cells were then incubated with FITC-conjugated anti-CD25 or anti-CD69 before analysis using flowcytometry as described in Materials and Methods. The results are from one representative from of three independent experiments.

**Fig. 4:**
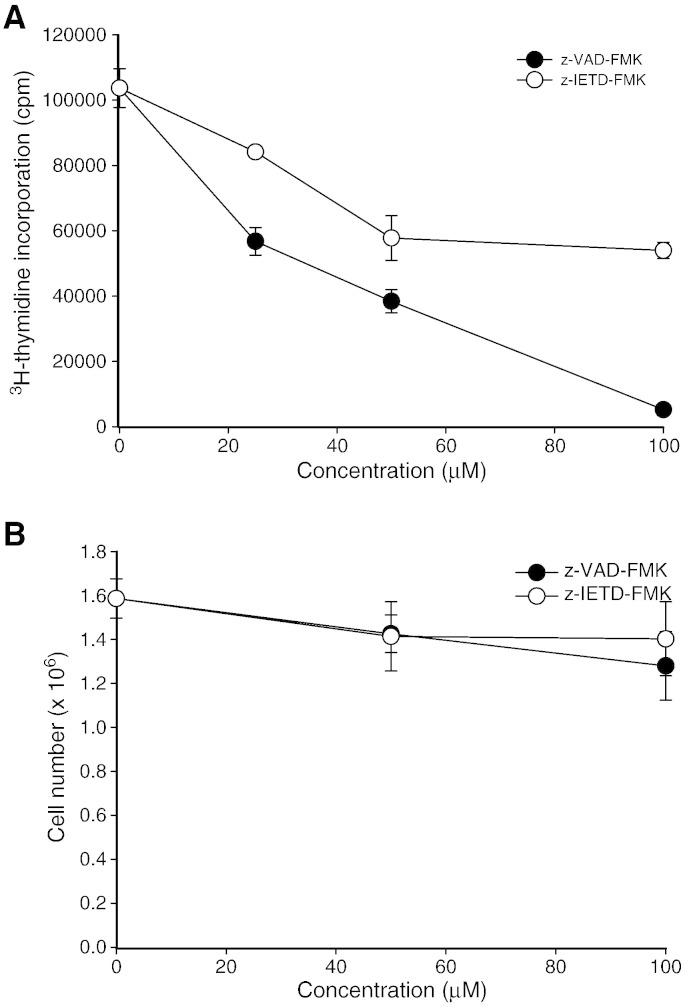
Effect of z-VAD-FMK and z-IETD-FMK on IL-2-driven T cell proliferation and normal Jurkat T cell growth. (A) Preactivated T cells were cultured in medium supplemented with rIL-2 in the absence or presence of the caspase inhibitors for 72 h. The incorporation of [^3^H]-thymidine was determined as outlined in Materials and Methods. Results are the means of one representative experiment out of three. (B) Jurkat T cells (2.5 × 10^5^ cells/ml) were incubated in the presence or absence of caspase inhibitors for 72 h. The results are the means ± SEM from three separate experiments.

Following T cell activation, IL-2 is synthesised and secreted, which subsequently stimulates T cells in an autocrine and paracrine fashion to drive T cell proliferation (Nelson, 2004). To determine the underlying mechanism of the caspase inhibitor-mediated inhibition of mitogen-induced T cell proliferation, we examined whether IL-2 secretion was affected. As shown in Fig. 2A, control untreated cells secrete little IL-2, whereas following co-stimulation with anti-CD3 and anti-CD28 there was a marked increase in IL-2 secretion into the culture supernatant as detected using ELISA. Neither z-VAD-FMK nor z-IETD-FMK had any significant effect on IL-2 secretion following T cell activation. We next determined whether these two caspase inhibitors had any effect on IFN-γ secretion following T cell activation. As illustrated in Fig. 2B, similar to IL-2 secretion, both z-VAD-FMK and z-IETD-FMK had no significant effect on the production of IFN-γ in activated T cells. We next examined whether the up-regulation of the α-subunit of the IL-2 receptor (CD25) is affected by these caspase inhibitors. Since T cell proliferation following activation is IL-2 driven, a decrease in CD25 will ultimately decrease cell proliferation and division. As shown in Fig. 3, the percentage of cells that stained positive for CD25 expression increased from around 4% in the control untreated cells to approximately 60% following activation with anti-CD3 plus anti-CD28. In the presence of z-VAD-FMK the up-regulation of CD25 was reduced to 46% and 31% at 50 μM and 100 μM, respectively. z-IETD-FMK was slightly less effective, reducing the percentage of activated T cells expressing CD25 to 52% and 35% at 50 μM and 100 μM, respectively. However, both caspase inhibitors had little effect on the expression of CD69, an early T cell marker which is stored preformed in the cytoplasm prior to expression on the cell surface (Risso et al., 1991). These findings suggest that both of these peptidyl-FMK inhibitors may render the cells unresponsive to IL-2 through the inhibition of CD25 expression. To examine this, the effect of the peptidyl-FMK inhibitors on IL-2 driven T cell proliferation was determined. Purified primary T cells were activated for 7 days, washed and the proliferating T cells cultured in medium supplemented with rIL-2 (25 U/ml) in the presence of the peptidyl-FMK inhibitors. In this approach the cycling T cells already express high level of IL-2R on the cell surface; hence the presence of rIL-2 should drive T cell proliferation. As illustrated in Fig. 4A the control cycling T cells in the presence of rIL-2 continued to proliferate as shown by the uptake of [^3^H]-thymidine. In the presence of z-VAD-FMK the uptake of [^3^H]-thymidine was inhibited in a dose-dependent manner whereas z-IETD-FMK was less effective. Our results suggest that antigen and IL-2 driven T cell proliferation are sensitive to the caspase inhibitors. We next examined whether these two peptidyl-FMK have any effect on normal cell growth in a T cell line that do not require activation signal to drive proliferation. To this end, the T cell leukemia cell line, Jurkat was cultured in the presence of these caspase-inhibitors. As shown in Fig. 4B, both peptides have no effect on Jurkat cell growth suggesting that the caspase inhibitors maybe targeting activation signals leading to cell proliferation.

### Peptidyl-FMK caspase inhibitors blocked the nuclear translocation of NF-κB RelA (p65) in activated T lymphocytes

**Fig. 5:**
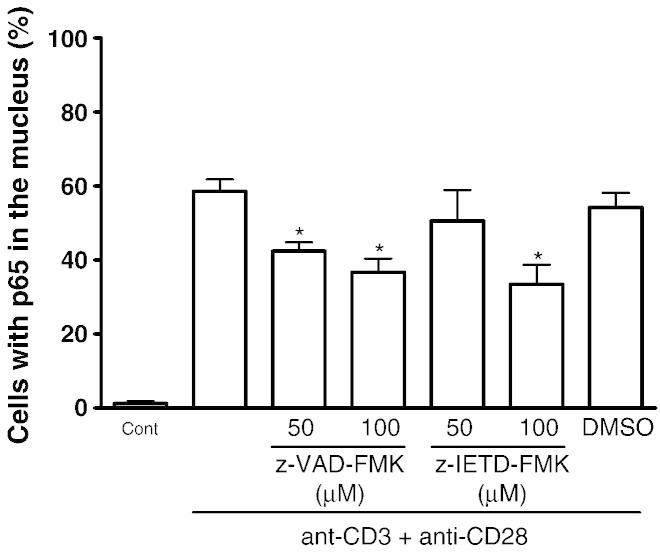
The effect of z-VAD-FMK and z-IETD-FMK on NF-kB signalling in activated primary T cells. Purified T cells were co-stimulated with anti-CD3 and anti-CD28 in the presence or absence of the caspase inhibitors for 2 h. The translocation of cellular p65 to the nucleus was determined as outlined in Materials and Methods. Results are the means ± SEM from three separate experiments. * Significantly different (p < 0.05) from cells co-activated with anti-CD3 and anti-CD28 alone. DMSO was used as the carrier solvent.

Because NF-κB is a well characterised transcription factor that is required for IL-2, IFN-γ and CD25 gene transcription as well as IL-2 signalling and T cell activation (Mortellaro et al., 1999), we examined the effect of the caspase inhibitors on the signalling of this transcription factor. The nuclear translocation of p65 (RelA) following TCR activation was examined using immunohistochemistry to localise p65 as previously reported (Lawrence et al., 2006). Following activation with anti-CD3 plus anti-CD28 for 2 h, the translocation of RelA into the nucleus was detected in ~ 58% of the activated T cells (Fig. 5) indicating that the NF-κB signalling was activated. In the presence of z-VAD-FMK (50 μM and 100 μM), there was a significant decrease in nuclear translocation of p65 in activated T cells, whereas only 100 μM z-IETD-FMK significantly inhibited p65 translocation. Taken together, these data suggest that the peptidyl-FMK caspase inhibitors inhibit NF-κB activation, which to some extent helps to explain the inhibition of T cell activation and proliferation, CD25 expression and IL-2 driven T cell proliferation.

### Time dependent processing of caspase-8 and caspase-3 during primary T cell activation

**Fig. 6:**
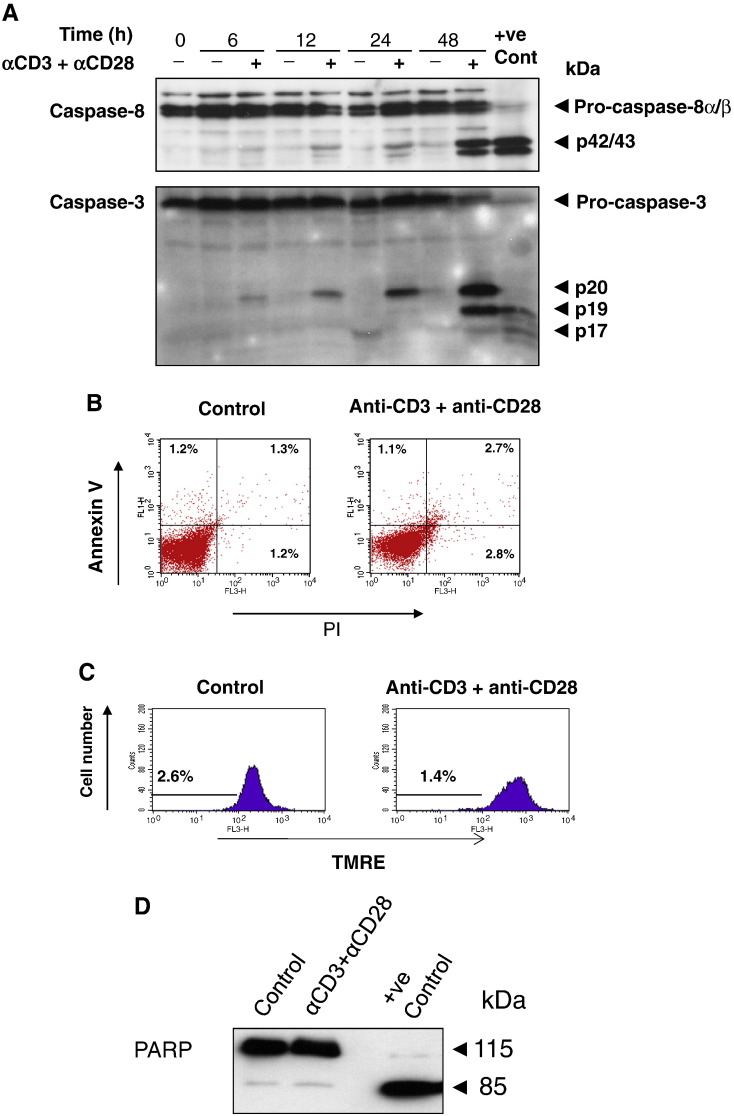
Caspase-8 and caspase-3 processing in newly activated T cells without apoptotic phenotype. (A) Purified T cells were co-stimulated with anti-CD3 and anti-CD28 for 6, 12, 24 and 48 h. Whole cell lysates were prepared and 30 μg of protein from each time point was resolved on 13% SDS-PAGE. The separated proteins were transferred to nitrocellulose membrane and probed for caspase-8 and caspase-3 as outlined in Materials and Methods. Following 24 h co-treatment with anti-CD3 and anti-CD28 the cells were processed for Annexin V staining and PI uptake (B), MMP stained with TMRE (C) PARP cleavage (D). FasL-treated Jurkat T cells were used as positive control (+ ve). All results are one representative from at least three independent experiments.

Previous studies have implicated the blocking of T cell proliferation by caspase inhibitors via the inhibition of caspases (Alam et al., 1999; Boissonnas et al., 2002; Falk et al., 2004; Kennedy et al., 1999; Mack and Hacker, 2002). To examine this we first determined the time course for caspase-8 and caspase-3 activation in T cells co-stimulated with anti-CD3 plus anti-CD28. As illustrated in Fig. 6A, no caspase-8 or caspase-3 processing was observed in resting primary T cells. However, following co-stimulation with anti-CD3 plus anti-CD28, a time-dependent processing of caspase-8 and caspase-3 into their respective intermediate subunits of p42/p43 and p20 was observed after 12 h. By 48 h caspase-8 and caspase-3 were further processed and the p20 subunit of caspase-3 was further cleaved to the p19 fragment. Based on these results the 24 h time-point was chosen for subsequent experiments. Since caspase processing is synonymous with apoptosis, several assays were used to rule out apoptosis in these activated T cells. As depicted in Fig. 6B, neither the control nor the activated T cells stained positive with FITC-conjugated annexin V, suggesting that the activated T cells were not apoptotic. The nuclei of these activated T cells remained normal without any apoptotic nuclei characteristics (nuclear condensation) following Hoechst dye staining (results not shown) and the cells had an intact mitochondrial membrane potential (Fig. 6C) as determined by TMRE staining of the mitochondrial membrane potential (Jayaraman, 2005; Johnson et al., 2000). Finally, the caspase-3 substrate, PARP which is cleaved during apoptosis, (Kaufmann et al., 1993) remained intact in these activated T cells (Fig. 6D). Taken together, these data demonstrated that the activation of caspase-8 and caspase-3 in activated T cells following activation was not due to the induction of apoptosis.

### z-VAD-FMK and z-IETD-FMK have no effect on the processing of caspase-8 and caspase-3 during primary T cell activation

**Fig. 7:**
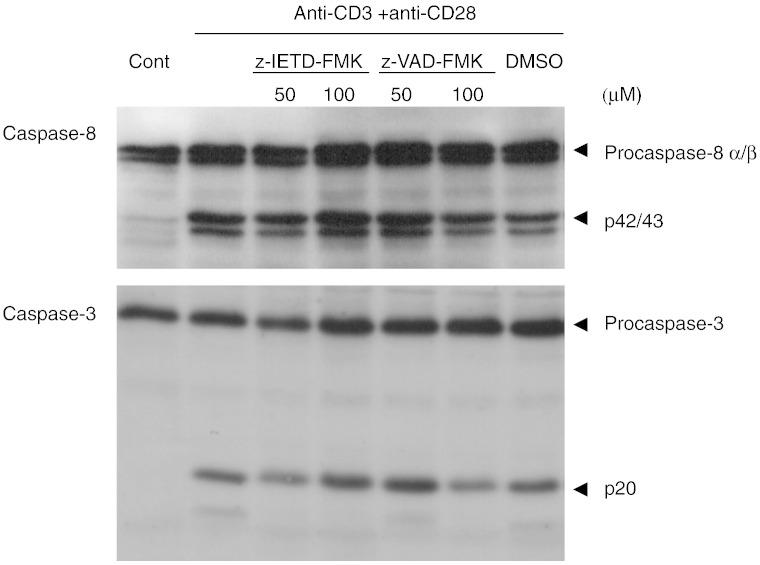
Effect of peptidyl FMK inhibitors on caspase processing in activated T cells. Purified T cells were co-activated with anti-CD3 and anti-CD28 for 24 h in the presence of z-VAD-FMK or z-IETD-FMK. Whole cell lysates (20 μg protein) were resolved on 13% SDS-PAGE and transferred to nitrocellulose membrane prior to probing for caspase-8 and caspase-3 as outlined in Materials and Methods. Results are one representative from three independent experiments. DMSO was used as the carrier solvent.

**Fig. 8:**
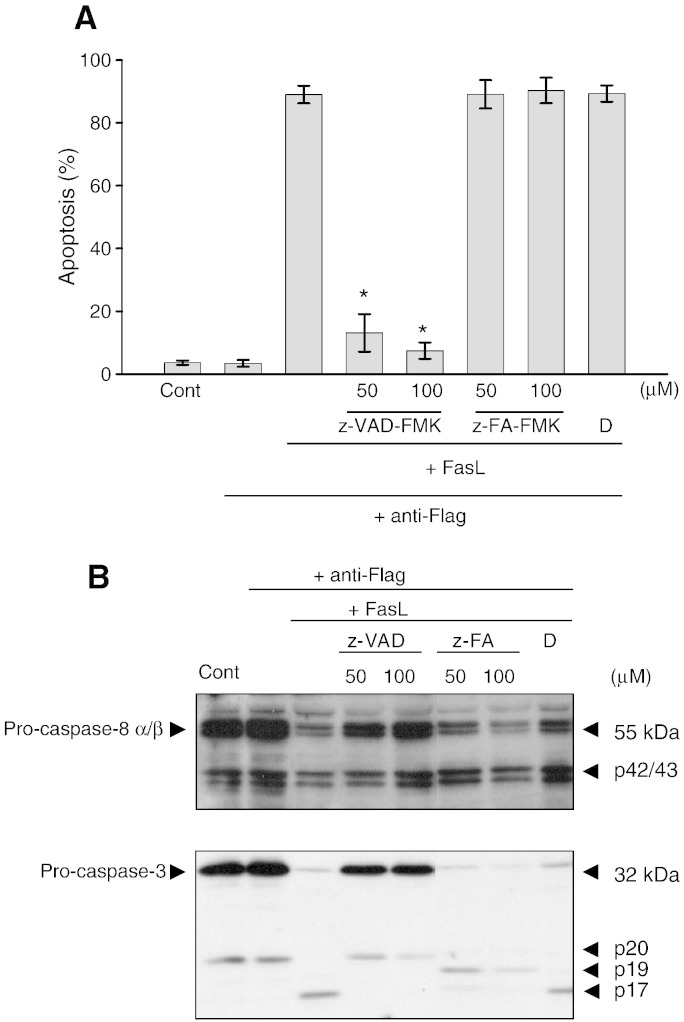
Effect of peptidyl FMK inhibitors on FasL-induced apoptosis and caspase-8 and caspase-3 processing in activated T cells. Activated T cells were treated with Flag-tagged FasL followed by cross-linking with anti-Flag for 16 h in the presence of z-VAD-FMK (z-VAD) or z-FA-FMK (z-FA). Apoptosis (A) and Western blot analysis (B) were carried out as outlined in Materials & Methods. The results for apoptosis are the means + SEM from three independent experiments and the immunoblots are are one representative out of three independent experiments. *Significantly decreased (*p* < 0.05) compared to cells treated with FasL alone. DMSO was used as the carrier solvent.

**Fig. 9:**
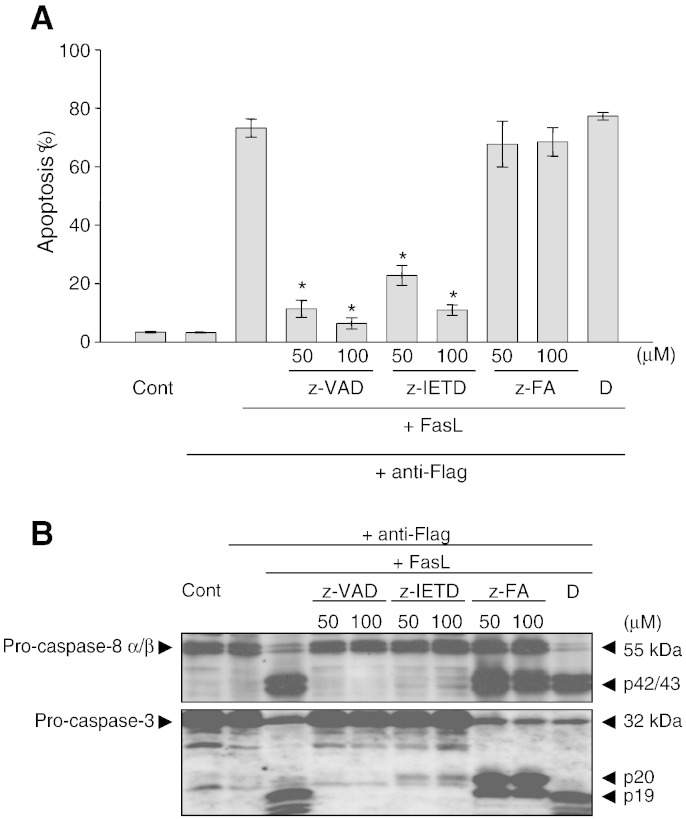
Effect of peptidyl FMK inhibitors on FasL-induced apoptosis and caspase-8 and caspase-3 processing in Jurkat T cells. Jurkat T cells were treated with Flag-tagged FasL followed by cross-linking with anti-Flag for 16 h in the presence of z-VAD-FMK (z-VAD), z-IETD-FMK (z-IETD) or z-FA-FMK (z-FA). Apoptosis (A) and Western blot analysis (B) were carried out as outlined in Materials & Methods. The results for apoptosis are the means ± SEM from three independent experiments and the immunoblots are are one representative out of three independent experiments. *Significantly decreased (*p* < 0.05) compared to cells treated with FasL alone. DMSO (D) was used as the carrier solvent.

Although previous studies have shown that both caspase inhibitors readily blocked T cell proliferation, it is not clear whether the activation of caspases during T cell activation is inhibited (Alam et al., 1999; Boissonnas et al., 2002). To examine this, purified resting T cells were pre-treated for 30 min with various concentrations of z-VAD-FMK or z-IETD-FMK prior to co-stimulation with anti-CD3 plus anti-CD28. As shown in Fig. 7, the western blot analysis showed that neither z-VAD-FMK nor z-IETD-FMK up to 100 μM had any effect on the activation of caspase-8 following T cell activation as shown by the presence of p42/43 cleaved intermediates. Similarly, both caspase inhibitors have little effect on the processing of caspase-3 to the p20 subunit, although they partially inhibited the processing of the p20 subunit to the smaller fragments. These results demonstrated that both caspase inhibitors have no effect on the activation of caspase-8 and caspase-3 in T cells following co-stimulation with anti-CD3 and anti-CD28. To confirm that z-VAD-FMK and z-IETD-FMK block caspase activity, we examined their effects on caspase processing in activated primary T cells (Fig. 8) and Jurkat T cells (Fig. 9) undergoing FasL-mediated apoptosis. As shown in Fig. 8A, activated T cells undergo apoptosis readily when treated with FasL for 16 h which was effectively blocked by z-VAD-FMK (50 and 100 μM). As expected, western blot analysis showed that some caspase-8 and caspase-3 were processed in control activated T cells (Fig. 8B), and more were processed to their respective subunits, p42/43 and p19/17 during FasL-induced apoptosis. The presence of z-VAD-FMK partially inhibited the processing of caspase-8 and caspase-3, suggesting that it may be blocking the caspases that were activated during apoptosis and not those processed during cell activation. In contrast, z-FA-FMK which is a control for z-VAD-FMK hardly has any effect on FasL-induced cell death and the activation of caspase-8 and caspase-3 in activated T cells. Similarly, both z-VAD-FMK and z-IETD-FMK inhibited FasL-induced apoptosis and blocked the activation of caspase-8 and caspase-3 in Jurkat T cells, whereas z-FA-FMK has little effect (Figs. 9A & B). Taken together, these data suggest that z-VAD-FMK and z-IETD-FMK inhibit caspase processing during apoptosis but not during T cell activation. In contrast, z-FA-FMK has no effect on caspase processing during apoptosis and did not block FasL-induced apoptosis in activated T cells and Jurkat T cells.

## Discussion

The role of caspases, in particular caspase-8, during T cell activation and proliferation is now well established, although their function in regulating proliferation is still unclear. Some of the earliest evidence to support caspase involvement in T cell proliferation came from studies using peptidyl-FMK caspase inhibitors. These compounds were shown to markedly reduce mitogen-induced T cell proliferation, suggesting that caspase enzymatic activity is required for T cell activation and proliferation (Alam et al., 1999; Boissonnas et al., 2002; Kennedy et al., 1999; Mack and Hacker, 2002) (Falk et al., 2004). However, accumulating evidence suggests that the peptidyl-FMK caspase inhibitors, which have been widely used in apoptosis research, may be associated with non-specific effects (Deszcz et al., 2004; Misaghi et al., 2006; Schotte et al., 1999). In the present study, we examined whether the inhibition of mitogen-induced T cell proliferation by the broad-spectrum caspase inhibitor, z-VAD-FMK and the caspase-8 selective inhibitor, z-IETD-FMK is mediated through the inhibition of caspases.

In agreement with several reports (Alam et al., 1999; Boissonnas et al., 2002; Falk et al., 2004; Kennedy et al., 1999; Mack and Hacker, 2002), we showed that mitogen-induced T cell proliferation was readily inhibited by z-VAD-FMK and z-IETD-FMK. Besides antigen induced T cell proliferation, IL-2 driven T cell proliferation was also inhibited by these two caspase inhibitors although z-IETD-FMK was less effective compared with z-VAD-FMK. In addition to blocking T cell proliferation, these compounds were found to reduce the expression of CD25, an early T cell activation marker which requires gene transcription. Together with CD25, a wide variety of genes that control immune responses are regulated by the NF-κB family of transcription factors. The NF-κB complexes are localised in the cytoplasm in resting T cells, where they are bound to inhibitor proteins (IκBs). In T cells the predominant form of NF-κB complexes that are activated during T cell activation is a heterodimer of the p65 subunit associated with either p50 or p52 subunits, although xRel/p50 is also present (Grilli et al., 1993; Tak and Firestein, 2001). Once activated, the inhibitory proteins, IκB are rapidly phosphorylated and degraded, which in turn releases the NF-κB transcription factors to be translocated into the nuclei and together with AP-1, regulate transcription (Grilli et al., 1993). In agreement with a previous study (Su et al., 2005) as well as our own (Lawrence et al., 2006), a large number of primary T cells activated through the antigen receptor were stained positive for p65 in the nucleus. In the presence of the caspase inhibitors, the nuclear translocation of p65 in activated primary T cells was significantly reduced, suggesting that NF-κB signalling induced by antigen receptor stimulation is suppressed. This could account for the reduced expression of CD25 since NF-κB regulated gene transcription is known to be required for this process. In addition, the activation of NF-κB is also required for IL-2 signalling (Mortellaro et al., 1999), which could explain the inhibition of rIL-2 driven T cell proliferation in the presence of z-VAD-FMK and z-IETD-FMK. However, neither z-VAD-FMK nor z-IETD-FMK inhibited IL-2 or IFN-γ secretion, which is unexpected since NF-kB signalling is also required for the transcription of these two cytokines (Aronica et al., 1999; Hentsch et al., 1992). One explanation for this could be insufficient inhibition of NF-κB signalling by these compounds. However, in addition to NF-κB signalling, antigen stimulated gene transcription is also regulated by other transcription factors such as NFAT and AP-1 (Hentsch et al., 1992; Luo et al., 1996). Therefore, it would be interesting to determine the effects of these peptidyl-FMK inhibitors on the activation of NFAT and AP-1 to reconcile these observations.

Besides promoting cell death, caspases have been shown to play an important role in T cell activation (Chun et al., 2002). We showed that following T cell activation through the antigen receptor, both caspase-8 and caspase-3 were activated in the cells and this was independent of any apoptotic characteristics. Surprisingly, both z-VAD-FMK and z-IETD-FMK had virtually no effect on the processing of caspase-8 and caspase-3 in these cells, which supports a previous study where Boc-D-FMK, a broad-spectrum caspase inhibitor, has no effect on caspase-3 processing during T cell activation (Bidere et al., 2002). Our findings suggest that the processing of caspase-8 and caspase-3 during T cell activation is mediated through a pathway which is insensitive to z-VAD-FMK or z-IETD-FMK and is unlikely to involve caspases. This is in contrast to FasL-induced apoptosis in Jurkat T cells where the processing of both caspase-8 and caspase-3 was effectively blocked by z-VAD-FMK and z-IETD-FMK. More importantly, we can infer from our results that the inhibition of antigen driven T cell activation and proliferation by z-VAD-FMK and z-IETD-FMK has little to do with the inhibition of caspase-8 and caspase-3 processing. Taken together, these results suggest that the processing of caspase-8 and caspase-3 during T cell activation is regulated by a distinct mechanism that is different from FasL-mediated apoptosis.

The finding that caspase-8 and caspase-3 was processed in activated T cells in the absence of apoptotic features, suggests that the apoptotic pathway must be inhibited at some stage downstream of caspase-8 and caspase-3 processing. In the present study, the caspase-3 substrate, PARP remained intact, suggesting that caspase-3 activity was held in check prior to the processing of PARP. This is in agreement with previous study where PARP was not cleaved in activated T cells (Deas et al., 1998). The finding that caspase-3 was only processed as far as the p20 subunit in activated T cells does not account for the lack of PARP cleavage, since removal of the N-terminal prodomain and thus generation of the p17 subunit from the p20 is not required for caspase-3 to cleave PARP (Stennicke et al., 1998). However, in contrast to the findings in this study, other studies have demonstrated PARP processing, in the absence of apoptotic features in activated T cells (Alam et al., 1999; Wilhelm et al., 1998). Therefore, the mechanism for the prevention of apoptosis, despite the presence of processed caspases remains to be determined.

In summary, the results presented here show that caspase processing in activated T cells is not inhibited by z-VAD-FMK or z-IETD-FMK. Since both z-VAD-FMK and z-IETD-FMK effectively inhibited T cell proliferation, but had minimal effects on caspase processing in activated T cells, it is unlikely that the inhibition of caspase processing is the means by which they exert their inhibitory effect. Indeed, it has recently been reported that z-VAD-FMK inhibits the enzymatically active proform of caspase-8 which is required for TCR-mediated NF-κB activation, rather than processed caspase-8 (Su et al., 2005). Further work is required to determine whether z-VAD-FMK inhibits pro-caspase-8 activity and whether z-IETD-FMK has a similar effect. The finding that z-FA-FMK inhibited caspase-8 and caspase-3 processing in activated T cells but did not inhibit caspases *per se* suggests that it inhibits an upstream mediator of caspase processing during T cell activation (Lawrence et al., 2006). Furthermore, the disparate effects of these peptidyl-FMK inhibitors on caspase-8 and caspase-3 processing during T cell activation and Fas-mediated apoptosis suggests that these processes are regulated by distinct mechanisms.

## Conflict of interest

The authors declare that there are no conflicts of interest.
